# ICE*Apl1*, an Integrative Conjugative Element Related to ICE*Hin1056*, Identified in the Pig Pathogen *Actinobacillus pleuropneumoniae*

**DOI:** 10.3389/fmicb.2016.00810

**Published:** 2016-06-15

**Authors:** Janine T. Bossé, Yanwen Li, Roberto Fernandez Crespo, Roy R. Chaudhuri, Jon Rogers, Matthew T. G. Holden, Duncan J. Maskell, Alexander W. Tucker, Brendan W. Wren, Andrew N. Rycroft, Paul R. Langford, the BRaDP1T Consortium

**Affiliations:** ^1^Section of Paediatrics, Department of Medicine, Imperial College LondonLondon, UK; ^2^Department of Veterinary Medicine, University of CambridgeCambridge, UK; ^3^Animal and Plant Health Agency Bury St EdmundsSuffolk, UK; ^4^The Wellcome Trust Sanger Institute, Wellcome Trust Genome Campus, HinxtonCambridge, UK; ^5^Faculty of Infectious and Tropical Diseases, London School of Hygiene and Tropical MedicineLondon, UK; ^6^Department of Pathology and Pathogen Biology, The Royal Veterinary CollegeHatfield, UK

**Keywords:** animal infections, antibiotic resistance, respiratory tract, conjugation, *Pasteurellaceae*

## Abstract

ICE*Apl1* was identified in the whole genome sequence of MIDG2331, a tetracycline-resistant (MIC = 8 mg/L) serovar 8 clinical isolate of *Actinobacillus pleuropneumoniae*, the causative agent of porcine pleuropneumonia. PCR amplification of *virB4*, one of the core genes involved in conjugation, was used to identify other *A. pleuropneumoniae* isolates potentially carrying ICE*Apl1*. MICs for tetracycline were determined for *virB4* positive isolates, and shotgun whole genome sequence analysis was used to confirm presence of the complete ICE*Apl1*. The sequence of ICE*Apl1* is 56083 bp long and contains 67 genes including a Tn*10* element encoding tetracycline resistance. Comparative sequence analysis was performed with similar integrative conjugative elements (ICEs) found in other members of the *Pasteurellaceae*. ICE*Apl1* is most similar to the 59393 bp ICE*Hin1056*, from *Haemophilus influenzae* strain 1056. Although initially identified only in serovar 8 isolates of *A. pleuropneumoniae* (31 from the UK and 1 from Cyprus), conjugal transfer of ICE*Apl1* to representative isolates of other serovars was confirmed. All isolates carrying ICE*Apl1* had a MIC for tetracycline of 8 mg/L. This is, to our knowledge, the first description of an ICE in *A. pleuropneumoniae*, and the first report of a member of the ICE*Hin1056* subfamily in a non-human pathogen. ICE*Apl1* confers resistance to tetracycline, currently one of the more commonly used antibiotics for treatment and control of porcine pleuropneumonia.

## Introduction

*Actinobacillus pleuropneumoniae* is a major contributor to swine respiratory disease worldwide, causing considerable economic losses. Isolates can be differentiated into 15 established serovars, based on capsular polysaccharides, and a recently proposed serovar 16 identified on the basis of serology alone (Sárközi et al., 2015). There are geographical differences in the distribution of serovars. Within the UK, clinical isolates are predominantly serovar 8, with serovars 2, 6, 7, and 12 also represented (O’Neill et al., 2010).

There is growing concern regarding antimicrobial resistance in bacteria from food-producing animals (Michael et al., 2015). In Europe, tetracyclines are still the most commonly used antimicrobial for treatment of swine pleuropneumonia (European Medicines Agency, 2012). The genes *tetB, tetH, tetL*, and *tetO*, reported to mediate tetracycline resistance in *A. pleuropneumoniae*, are usually carried on small plasmids (Blanco et al., 2006, 2007). We recently sequenced the genome of MIDG2331, a serovar 8 UK clinical isolate of *A. pleuropneumoniae* (Bossé et al., 2016), and identified chromosomally encoded tetracycline resistance genes within a putative integrative conjugative element (ICE). Similar to genomic islands, ICE have the ability to integrate into bacterial chromosomes at specific sites, often in tRNA loci, via the action of an integrase (predominantly tyrosine recombinases) (Boyd et al., 2009; Wozniak and Waldor, 2010). However, ICE differ from genomic islands in that they are self-mobilizing, encoding all of the genes necessary for excision from the chromosome and conjugal transfer (Boyd et al., 2009). The core genes of ICE tend to group into functional modules, with syntenic regions responsible for maintenance, dissemination and regulation, which may be interspersed with accessory genes carried on transposons or other insertion elements (Burrus and Waldor, 2004; Wozniak and Waldor, 2010). The genes encoding the type 4 secretion system (T4SS), required for transport of DNA into recipient cells, include a ubiquitous ATPase encoded by *virB4* or *traU* (Guglielmini et al., 2011).

ICEs are the most abundant conjugative elements identified in prokaryotes, and there is evidence of cross-clade transfer (Guglielmini et al., 2011). Within the *Pasteurellaceae*, ICE have been identified and characterized in *Haemophilus influenzae* and *Haemophilus parainfluenzae* (Juhas et al., 2007b), *Pasteurella multocida* (Michael et al., 2012), and *Mannheimia haemolytica* (Eidam et al., 2015). Here we report characterization of ICE*Apl1*, to our knowledge the first ICE described in *A. pleuropneumoniae*.

## Materials and Methods

### Comparative Sequence Analysis

The full sequence of ICE*Apl1*, identified within the genome of MIDG2331 (accession number LN908249) was analyzed using BLASTn and BLASTx^1^. A comparative alignment was generated for sequences most similar to ICE*Apl1* using Mauve version 2.3.1^2^. Default parameters were used for all programs.

### Detection of Other Isolates Containing ICE*Apl1*

We screened 185 isolates of *A. pleuropneumoniae* (clinical isolates collected between 1995 and 2012 from the UK, Denmark, the Czech Republic, Cyprus, and Greece) for *virB4* by PCR using primers virB4_for (CCTTCACGGTTAAAGAATCG AC)/virB4_rev (GCATCGTTTATTGGAAATGGAT). Primers were designed based on the *virB4* gene in MIDG2331, amplifying the region from 1532511 to 1532894 in the genome sequence. Serovars 1 (1.2%), 2 (11.7%), 5 (2.4%), 6 (4.7%), 7 (10.6%), 8 (58.8%), 9/11 (2.4%), 10 (2.9%), and 12 (5.3%), were represented, and 84% of the isolates were from the UK. Genome sequence data was generated and assembled as previously described (Howell et al., 2013; Bossé et al., 2015) for 31 *virB4* positive isolates. Sequences matching ICE*Apl1* were identified by BLASTn, assembled using Geneious 9.0.4, and deposited to Genbank (see **Table 1** for accession numbers).

**Table 1:** Clinical isolates of *Actinobacillus pleuropneumoniae* with ICE*Apl1*.

Isolate ID	Serovar	Location of isolation^a^	Year of isolation	Length of ICE*Apl1*^b^	5′ tRNAs^c^	Accession number for ICE*Apl1* sequence
MIDG2331	8	Thirsk	1995	56,083 bp	GLGLL	LN908249 (bases 1570419–1570505)^d^
MIDG2427	8	Aberdeen	1998	56,083 bp	GLGLL	KU551309
MIDG2648	8	Bury St Edmunds	2005	**54,898 bp**	**GLGL**	KU551310
MIDG2652	8	Thirsk	2005	56,083 bp	GLGLL	KU551311
MIDG2654	8	Winchester	2005	**56,070 bp**	GLGLL	KU551312
MIDG2657	8	Winchester	2005	56,083 bp	**GLLGL**	KU551313
MIDG2663	8	Thirsk	2005	56,083 bp	**GLLGL**	KU551314
MIDG2664	8	Bury St Edmunds	2005	56,083 bp	**GLLGL**	KU551315
MIDG3200	8	Thirsk	2006	56,083 bp	GLGLL	KU551316
MIDG3201	8	Bury St Edmunds	2006	56,083 bp	GLGLL	KU551317
MIDG3221	8	Langford	2006	56,083 bp	GLGLL	KU551318
MIDG3229	8	Thirsk	2007	56,083 bp	GLGLL	KU551320
MIDG3232	8	Thirsk	2007	56,083 bp	GLGLL	KU551321
MIDG3339	8	Winchester	2008	56,083 bp	GLGLL	KU551322
MIDG3344	8	Langford	2005	56,083 bp	**GLLGL**	KU551323
MIDG3346	8	Thirsk	2005	56,083 bp	**GLLGL**	KU551324
MIDG3349	8	Thirsk	2006	56,083 bp	**GLLGL**	KU551325
MIDG3357	8	Shrewsbury	2008	56,083 bp	GLGLL	KU551326
MIDG3368	8	Thirsk	2008	56,083 bp	GLGLL	KU551327
MIDG3370	8	Thirsk	2009	56,083 bp	**GLLGL**	KU551328
MIDG3371	8	Thirsk	2009	56,083 bp	**GLLGL**	KU551329
MIDG3372	8	Thirsk	2009	56,083 bp	**GLLGL**	KU551330
MIDG3378	8	Bury St Edmunds	2009	**56,047 bp**	GLGLL	KU551331
MIDG3381	8	Thirsk	2009	56,083 bp	GLGLL	KU551332
MIDG3386	8	Bury St Edmunds	2009	56,083 bp	GLGLL	KU551333
MIDG3388	8	Thirsk	2009	56,083 bp	GLGLL	KU551334
MIDG3389	8	Thirsk	2009	56,083 bp	GLGLL	KU551335
MIDG3395	8	Thirsk	2010	**56, 011 bp**	GLGLL	KU551336
MIDG3401	8	Bury St Edmunds	2011	56,083 bp	GLGLL	KU551337
MIDG3409	8	Bury St Edmunds	2011	56,083 bp	GLGLL	KU551338
MIDG3458	8	Cyprus	2011	**56,012 bp**	GLGLL	KU551339
MIDG3469	8	Thirsk	2012	56,083 bp	GLGLL	KU551340

*^a^All isolates except MIDG2427 and MIDG3458 were cultured from pigs submitted to the then Veterinary Laboratories Agency (now Animal and Plant Health Agency) regional laboratories in the UK, as indicated.*

*^b^The length of each ICE*Apl1* sequence, calculated as the predicted circular form, is shown for each isolate. Size variation from that in MIDG2331 is indicated in bold. The sequence in MIDG2648 is missing 3 genes and has a truncated copy of the site-specific recombinase gene; whereas small deletions in the other sequences are all intergenic.*

*^c^Order of tRNA genes upstream of ICE*Apl1*: G, tRNA-Gly (GCC), L, tRNA-Leu (TAA). Variation from the order seen in MIDG2331 is indicated in bold.*

*^d^ICE*Apl1* identified in MIDG2331 in previous study,^4^ all other ICE*Apl1* sequences were identified in this study.*

Minimum Inhibitory Concentrations (MICs) for tetracycline were determined for isolates containing ICE*Apl1*, according to the CLSI M37-A3 guidelines (Clinical and Laboratory Standards Institute [CLSI], 2008).

### Conjugal Transfer of ICE*Apl1*

MIDG2331Δ*ureC*::*nadV* was used as the conjugal donor, with matings performed as previously described (Bossé et al., 2015). Plasmid-free, tetracycline-sensitive, nalidixic acid-resistant clinical isolates of serovars 6 (MIDG3376), 7 (MIDG2465), 8 (MIDG3217), and 12 (MIDG3347) were used as recipients. Transconjugants were selected on Brain Heart Infusion agar supplemented with 0.01% NAD, 5 mg/L tetracycline and 40 mg/L nalidixic acid. PCR was used to confirm the presence of the *virB4* gene (as above), as well as serovar of, and the absence of *nadV*, in selected transconjugants using previously described primers (Bossé et al., 2014, 2015). Chromosomal insertion sites in transconjugants were determined by PCR using primers ICE5′_out1 (TGAGGGAGTAACAAGCAACACAG)/mfd3′_out (TTTACCGCTTGCCGATAATGCG) for the 5′ junction, and ICE3′_out1 (CAATGGAGAAAGAGAGTTGTTGGAC) /hybF5′_out (GACATCTCGTGCATAACCATTCC) for the 3′ junction, respectively. Amplicons were sequenced using internal primers ICE5′_out2 (GGAAGGTTCAATATCA CGACGG) or ICE3′_out2 (AGGCATACAGCAGCAACAAATC), as appropriate. For comparison, the region between *mfd* and *hybF* in the conjugal recipients (prior to conjugation) was amplified using mfd3′_out/hyb5′_out and sequenced in both directions.

### Confirmation of the Circular Extrachromosomal form of the ICE by Nested PCR

DNA was extracted from MIDG2331Δ*ureC*::*nadV* and selected transconjugants, and nested PCR was performed as previously described (Eidam et al., 2015), using primers ICE5′_out1/ICE3′_out1 followed by primers ICE5′_out2/ICE3′_out2. Amplicons were sequenced using primers ICE5′_out2 and ICE3′_out2.

## Results and Discussion

### Sequence of ICE*Apl1* and Comparative Analysis

**FIGURE 1:**
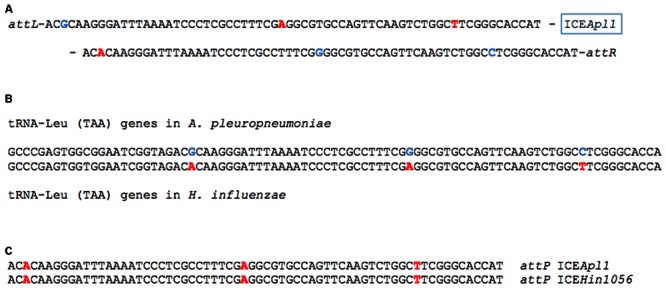
**Analysis of ICE*Apl1* insertion site.** The imperfect direct repeats (DRs) **(A)** flanking ICE*Apl1* share sequence identity with the last 65 bases of tRNA-Leu genes **(B)** from *Actinobacillus pleuropneumoniae* (e.g., MIDG2331_01482 and _01484) and *Haemophilus influenzae* (e.g., accession number LK008335), which differ at 3 bases (in light blue for bases normally found in *A. pleuropneumoniae* and red for bases normally found in *H. influenzae*). The *attI* sequences **(C)** in the closed circular forms of ICE*Apl1* and ICE*Hin1056* are identical, and match the end of the *H. influenzae* tRNA-Leu (TAA) gene sequence, with an additional T which is also present in the DRs **(A)**. Insertion of ICE*Apl1* in the *A. pleuropneumoniae* tRNA-Leu (TAA) gene MIDG2331_01481 has resulted in an altered sequence, indicating cross-over of the circular ICE*Apl1* into the chromosome occurred between the G at position 25 and the A at position 54.

ICE*Apl1*, a 56083 bp element, is inserted into a tRNA-Leu (TAA) gene, a common insertion site for ICE in the family *Pasteurellaceae* (Dimopoulou et al., 2002; Michael et al., 2012; Eidam et al., 2015), in a tRNA cluster located between genes *hybF* and *mfd*. In the MIDG2331 genome (Bossé et al., 2016), this tRNA-Leu (TAA) gene is annotated as MIDG2331_01481, and is located between bases 1570419 and 1570505. Although all of the tRNA genes in the cluster, as well as *hybF* and *mfd*, are on the complement strand in the MIDG2331 genome, all further references to these genes, and the location of ICE*Apl1*, will be with respect to the forward orientation. Insertion of ICE*Apl1* generated 66 bp imperfect direct repeats (DRs) at the left and right attachment sites, *attL* and *attR* (**Figure 1A**). The three bases that differ in the DRs reflect sequence variation in the tRNA-Leu (TAA) genes in *A. pleuropneumoniae* and *H. influenzae* (**Figure 1B**). The *attI* site in the closed circular form of ICE*Apl1* (confirmed by PCR; see below) is identical to that in ICE*Hin1056* (**Figure 1C**), both having 100% identity with the last 65 bases of the *H. influenzae* tRNA-Leu (TAA) gene followed by a T. These data suggest that insertion of ICE*Apl1* in MIDG2331 has generated an altered tRNA-Leu (TAA) gene, resulting from cross-over of the circular plasmid form of the ICE into the chromosome between the G at position 25 and the A at position 54 of MIDG2331_01481.

**FIGURE 2:**
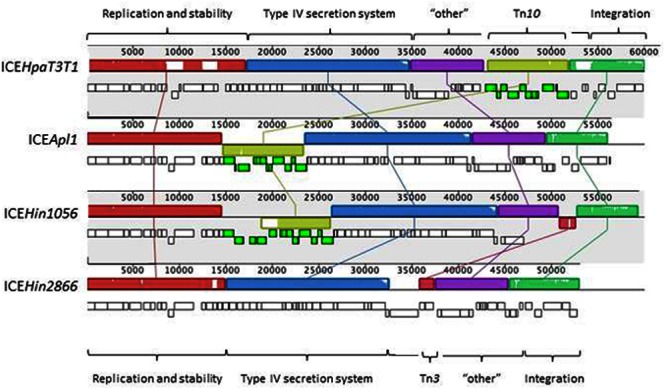
**Mauve alignments of ICE*Apl1* and closely related members of the ICE*Hin1056* subfamily of elements.** The orientation and relative size of genes in each ICE are indicated by the small rectangular blocks (lower blocks are on the complementary strand), with those colored green indicating genes of Tn*10* elements present in all except ICE*Hin2866*. Note the extra genes in the Tn*10* insertion in ICE*Hin1056* are due to the presence of a further IS*5* insertion not seen in the other ICE. Regions containing contiguous genes of related function are indicated by colored blocks above the genes, and are connected by lines to matching blocks in each ICE sequence. The labels at the top and bottom of the figure indicate functional sets of genes defined by Juhas et al. (2007b).

Comparative sequence analysis revealed that ICE*Apl1* is related to the ICE*Hin1056* subfamily of elements (**Figure 2**) found in *H. influenzae* and *H. parainfluenzae* (Mohd-Zain et al., 2004; Juhas et al., 2007b). ICE*Apl1* encodes 67 genes that share extensive sequence homology and gene order with ICE*Hin1056* and other members of this subfamily. The first 14687 bp of ICE*Apl1* shares 99% identity with the region of ICE*Hin1056* reported to contain replication and stabilization genes (Juhas et al., 2007a,b, 2013). The 8933 bp Tn*10* element in ICE*Apl1*, although in the same location and orientation as that in ICE*Hin1056*, more closely resembles that in ICE*HpaT3T1* (99% identity, but inverted), with tetracycline resistance genes *tetR, tetB, tetC*, and *tetD*; and *gltS* encoding glutamate permease (Juhas et al., 2007b). The Tn*10* element in ICE*Hin1056* has a further IS*5* insertion (encoding chloramphenicol resistance) within it that is not seen in ICE*Apl1* (Juhas et al., 2007b). The 20466 bp following the Tn*10* insertion shares 99% identity with the region containing genes encoding components of the type IV secretion system (required for conjugal transfer) in ICE*Hin1056* (Juhas et al., 2007a,b, 2013). This region is well conserved in all of the members of the ICE*Hin1056* family (Juhas et al., 2007a,b, 2013). The gene order in ICE*Apl1* remains syntenic with that of ICE*Hin1056* up to *traC*, where in ICE*Hin1056* there is a Tn*3* insertion (encoding beta-lactamase resistance) that is not found in ICE*Apl1*. From *traC* in ICE*Apl1*, the nucleotide sequence and gene order more closely resemble those in ICE*Hin2866* up to the site-specific tyrosine recombinase gene, which is the final gene in ICE*Apl1* on the *attR* side. This 8.5 kb region shares 98% identity with sequences in ICE*Hin2866*, and includes accessory genes encoding a type I restriction enzyme M subunit, and a transposon gamma-delta resolvase, as well as four hypothetical genes of unknown function (Juhas et al., 2007b).

Surprisingly, the ICE*Hin1056* subfamily of conjugative elements has previously only been reported in *H. influenzae* and *H. parainfluenzae*, two human species of *Haemophilus*, where they appear to be evolving by descent (Dimopoulou et al., 2007; Juhas et al., 2007b). To our knowledge, this is the first report of a member of the ICE*Hin1056* subfamily in a *Pasteurellaceae* species that infects livestock. There have been ICE reported for bovine isolates of *P. multocida (*ICE*Pmu1*) and *M. haemolytica* (ICE*Mh1*), as well as an uncharacterized putative ICE in *Histophilus somni* strain 2336, which are related and appear to have evolved from a common ancestor, but are part of a different subfamily than ICE*Hin1056* elements (Juhas et al., 2007b; Michael et al., 2012; Eidam et al., 2015). The identity of the *attI* sites in both ICE*Apl1* and ICE*Hin1056* with the last 65 bases of the *H. influenzae* tRNA-Leu (TAA) gene would suggest more recent acquisition of an ICE*Hin1056* element in *A. pleuropneumoniae*.

### Distribution of ICE*Apl1* in *A. pleuropneumoniae* Isolates

PCR analysis revealed the presence of a *virB4* amplicon in 32/185 *A. pleuropneumoniae* isolates, including MIDG2331. All 32 (31 from the UK, 1 from Cyprus) were serovar 8 and had an MIC for tetracycline of 8 mg/L, i.e., above the CLSI breakpoint of ≥ 2 mg/L for *A. pleuropneumoniae* (Clinical and Laboratory Standards Institute [CLSI], 2008). ICE*Apl1* sequences were detected in the whole genomes of the 32 isolates, and comparative analysis revealed that, other than minor nucleotide differences in some, all of the sequences were complete except the element from MIDG2648, which was lacking three genes (encoding a putative DNA-binding protein and two hypothetical proteins) in the accessory gene region, and has a truncated copy of the tyrosine recombinase gene (**Table 1**).

### Conjugal Transfer of ICE*Apl1* and Detection of Circular Intermediate Form

As ICE*Apl1* appeared to be present only in serovar 8 isolates of *A. pleuropneumoniae*, it was possible that other serovars blocked conjugal entry of the ICE, either due to restriction modification systems or CRISPR mediated restriction (Elhai et al., 1997; Garneau et al., 2010). We therefore tested the ability to conjugally transfer ICE*Apl1* to clinical isolates of *A. pleuropneumoniae* representing serovars 6, 7, 8, and 12 (MIDG3376, MIDG2465, MIDG3217, and MIDG3347, respectively) that are commonly found in the UK (O’Neill et al., 2010). All tested isolates produced transconjugants, as initially confirmed by PCR (data not shown). The frequencies of conjugation were similar for the serovar 7, 8, and 12 recipients (between 10^-4^ and 10^-5^), but much lower (5 × 10^-8^) for the serovar 6 isolate tested. Similar frequencies have been reported for ICE*Hin1056* elements in *H. influenzae*, with strain related differences also noted (Juhas et al., 2007b). More serovar 6 isolates would need to be tested in order to determine if the difference in frequency of conjugation for ICE*Apl1* is serovar-specific. The presence of a circular intermediate form of ICE*Apl1* was confirmed in the donor strain and in transconjugants by nested PCR. Sequenced amplicons confirmed a single copy of the 66 bp *attI* (**Figure 1C**) at the closed junction of the circular intermediates.

**FIGURE 3:**
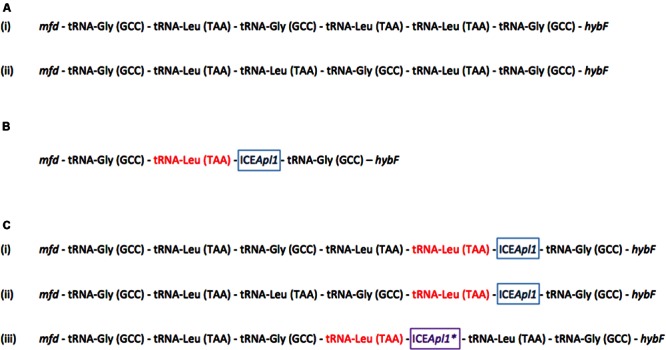
**Schematic representation of the tRNA cluster located between *mfd* and *hybF* in *A. pleuropneumoniae* isolates.** Note that all sequences are shown in the forward orientation for simplicity. In the MIDG2331 genome, these sequences are on the complement strand. **(A)** The tRNA genes in isolates used as conjugal recipients are in the same order in (i) MIDG2465 (serovar 7), MIDG3217 (serovar 8), and MIDG3347 (serovar 12); and a different order in (ii) MIDG3376 (serovar 6). Note that all of the tRNA-Leu (TAA) genes have the sequence shown in **Figure 1B**. **(B)** Following conjugation, ICE*Apl1* integration resulted in loss of 3 tRNA genes, with all of the transconjugants (MIDG2465::ICE*Apl1*, MIDG3217::ICE*Apl1*, MIDG3347::ICE*Apl1*, and MIDG3376::ICE*Apl1*) showing identical sequences flanking the insertion (ICE*Apl1* shown boxed in blue). Note that the tRNA-Leu (TAA) gene shown in red has the altered bases of the *attL* DRs in **Figure 1A**. **(C)** In the 32 isolates with endogenous ICE*Apl1*, the tRNA genes are found in three different orders in (i) MIDG2331 and 21 other isolates; (ii) 9 other isolates; and (iii) MIDG2648. Note the truncated element in MIDG2648 is shown as ICE*Apl1*^∗^ (in purple text, boxed in purple). See **Table 1** for details of specific isolates. Again, the tRNA-Leu (TAA) gene shown in red has the altered bases of the *attL* DRs in **Figure 1A**.

Sequencing PCR products generated at both the *attL* and *attR* ends in the transconjugants confirmed insertion of ICE*Apl1* in the same tRNA cluster between *mfd* and *hybF* as in the donor strain (MIDG2331Δ*ureC*::*nadV*). However, in all transconjugants tested, the sequences between *mfd* and *hybF* contained only the altered tRNA-Leu (TAA) gene in which the ICE inserted, flanked by tRNA-Gly (GCC) genes at either end of the cluster (**Figure 3B**). This is in contrast to the cluster in the donor strain where there are 5 tRNA genes on the *attL* side, and a tRNA-Gly (GCC) gene on the *attR* side (**Figure 3C**). Sequencing across the tRNA cluster in the recipient strains prior to conjugation revealed that, although a different order of tRNA genes was present in MIDG3376 compared to the other strains (**Figure 3A**), all contained 3 copies each of tRNA-Gly (GCC) and tRNA-Leu (TAA) genes. These results indicate that in all transconjugants tested, a deletion of 3 tRNA genes was associated with ICE*Apl1* insertion. However, examination of the insertion sites in the serovar 8 clinical isolates with endogenous ICE*Apl1* (**Table 1**) revealed conservation of the 6 tRNA genes normally found in this cluster – i.e., 3 copies each of tRNA-Gly (GCC) and tRNA-Leu (TAA) – with the order of genes showing one of 3 patterns (see **Figure 3C** and **Table 1** for details). In MIDG2648, the truncated element present in this isolate is located in a different copy of the tRNA-Leu (TAA) gene than seen in isolates with intact ICE*Apl1* (**Figure 3C**), with 4 tRNA genes on the *attL* side, and a tRNA-Leu (TAA) followed by tRNA-Gly (GCC) gene on the *attR* side. It would appear that, although the majority of clinical isolates show integration in the same tRNA-Leu (TAA) gene (i.e., the final copy in the tRNA cluster in the forward orientation), ICE*Apl1* has the ability to integrate into different copies of this gene, as has been reported for ICE*Hin1056* in *H. influenzae* (Dimopoulou et al., 2002). In *Pseudomonas knackmussii*, ICE*clc* was found to insert into different copies of the tRNA-Gly (GCC) gene, with double integration in some transconjugants (Sentchilo et al., 2009). In that study, excision and reintegration was associated with generation of a heterogenous population in which ICE*clc* was found to move from its original insertion site to alternate tRNA-Gly genes, but only those with the GCC anticodon (Sentchilo et al., 2009). Similarly, following *in vitro* conjugal transfer of ICE*Kp1* into a recipient strain of *Klebsiella pneumoniae*, integration was found at any of four tRNA-Asn genes, with insertion in multiple copies in some transconjugants (Lin et al., 2008). Furthermore, in some transconjugants, there was evidence of deletions between copies of the tRNA-Asn genes associated with integration of ICE*Kp1*, which may have been due to recombination between multiple insertion sites (Lin et al., 2008). We did not detect multiple insertions of ICE*Apl1* in our transconjugants, as only one PCR product was generated using the outward facing primers designed to amplify the closed junction of the circular ICE. These primers would also have generated a secondary product in the presence of tandem insertions, given the proximity of the copies of the tRNA-Leu (TAA) genes in *A. pleuropneumoniae*. It is also possible that recombination may occur between the DRs found in alternate copies of the target tRNA, with or without the presence of an integrated ICE, resulting in deletion of the intervening sequences. However, given the results of Lin et al. (2008), it is likely that conditions during *in vitro* conjugation favor integration of multiple copies of ICE leading to deletions, whereas this does not appear to be common amongst clinical isolates. Little is known regarding the signals that govern initiation of horizontal transfer of ICE between pathogens in a host animal environment.

## Conclusion

Identification of ICE*Apl1* in only serovar 8 clinical isolates of *A. pleuropneumoniae* may simply be a reflection of this being the most common in the UK (O’Neill et al., 2010), and thus in our collection. It may also indicate a tendency for ICE to be inherited by vertical transmission rather than horizontal transfer. The similar *in vitro* conjugation frequencies of ICE*Apl1* into isolates of serovars 7, 8, and 12 suggests there are no restriction endonuclease or CRISPR barriers to transfer between these serovars. Futhermore, the variation in order of tRNA genes flanking ICE*Apl1* suggests that horizontal transfer may have occurred independently into different isolates, as a similar variation in tRNA gene order was also seen in clinical isolates lacking ICE*Apl1*. As reported for other ICE, ICE*Apl1* has the ability to integrate into different copies of the target tRNA gene, in this case tRNA-Leu (TAA). Although we did not detect multiple insertions following *in vitro* conjugal transfer of ICE*Apl1*, it is likely that recombination between tandem insertions was responsible for the deletions detected in transconjugants.

To our knowledge, this is the first description of an ICE identified in *A. pleuropneumoniae*, and the first report of a member of the ICE*Hin1056* subfamily found in a non-human pathogen. The presence of ICE*Apl1* in isolates of *A. pleuropneumoniae* confers resistance to tetracycline, which is commonly used for treatment and control of porcine pleuropneumonia (European Medicines Agency, 2012). Although currently only found in serovar 8 isolates, the ability to transfer to other serovars was confirmed *in vitro*, and has implications for the spread of antimicrobial resistance in this important pig pathogen.

## Author Contributions

JB, PL, AR, BW, DM, and AT conceived the study; JB, YL, RFC, RRC, MH, and JR produced the data; JB, YL, RFC, and RRC analyzed the data; JB and PL wrote the paper.

## Conflict of Interest Statement

The authors declare that the research was conducted in the absence of any commercial or financial relationships that could be construed as a potential conflict of interest.

## Footnotes

1 http://blast.ncbi.nlm.nih.gov/

2 http://darlinglab.org/mauve/mauve.html
